# Plasmon-activated water effectively relieves hepatic oxidative damage resulting from chronic sleep deprivation†

**DOI:** 10.1039/c7ra13559a

**Published:** 2018-03-05

**Authors:** Hsiao-Chien Chen, Chung-Yi Cheng, Li-You Chen, Chun-Chao Chang, Chih-Ping Yang, Fu-Der Mai, Wen-Chieh Liao, Hung-Ming Chang, Yu-Chuan Liu

**Affiliations:** Department of Biochemistry and Molecular Cell Biology, School of Medicine, College of Medicine, Taipei Medical University 250 Wuxing St. Taipei 11031 Taiwan liuyc@tmu.edu.tw; Division of Nephrology, Department of Internal Medicine, Taipei Medical University Wan Fang Hospital 111 Hsing-Long Rd., Sec. 3 Taipei 116 Taiwan; Department of Anatomy, School of Medicine, College of Medicine, Chung Shan Medical University 110 Sec. 1, Jianguo N. Rd. Taichung 40201 Taiwan; Division of Gastroenterology and Hepatology, Department of Internal Medicine, School of Medicine, College of Medicine, Taipei Medical University and Taipei Medical University Hospital 250 and 252 Wuxing St. Taipei 11031 Taiwan; Department of Anatomy and Cell Biology, School of Medicine, College of Medicine, Taipei Medical University 250 Wuxing St. Taipei 11031 Taiwan taiwanose@tmu.edu.tw

## Abstract

The role of the hepato-protective agent plasmon-activated water (PAW) as an innovative anti-oxidant during chronic sleep deprivation (SD) is realized in this study. PAW possesses reduced hydrogen-bonded structure, higher chemical potential and significant anti-oxidative properties. *In vitro* tests using rat liver cell line (Clone-9) have demonstrated that PAW is non-cytotoxic and does not change the cellular migration capacity. The *in vivo* experiment on SD rats suffering from intense oxidative damage to the liver, an extremely common phenomenon in the present-time with deleterious effects on metabolic function, is performed by feeding PAW to replace deionized (DI) water. Experimental results indicate that PAW markedly reduces oxidative stress with enhanced bioenergetics in hepatocytes. PAW also effectively restores hepatocytic *trans*-membrane ion homeostasis, preserves membranous structures, and successfully improves liver function and metabolic activity. In addition, the hepato-protective effects of PAW are evidently demonstrated by the reduced values of glutamic oxaloacetic transaminase (GOT) and glutamic pyruvic transaminase (GPT) and the recovery of total protein and albumin levels. With clear evidences of PAW for protecting liver from SD-induced injury, delivering PAW as a powerful hepato-protective agent should be worthy of trailblazing new clinical trials in a healthier, more natural, and more convenient way.

## Abstract 2

Intrinsic anti-oxidative activity of PAW protects the liver from CSD-induced oxidative damage.

## Graphical abstract

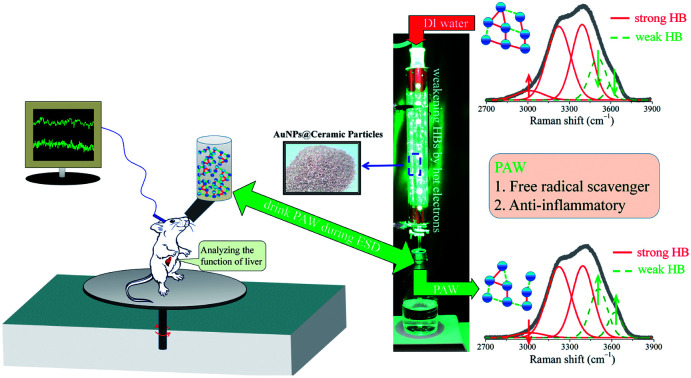

## 1. Introduction

In modern society, sleep deprivation (SD) has increasingly become a major public health issue, which affects millions of people in many countries worldwide.^1,2^ Chronic sleep loss can disturb the cellular function, which unavoidably leads to a variety of neurobiological, cardiovascular, and metabolic diseases.^3–5^ Previous studies have indicated that SD can significantly impair neuroendocrine bioenergetics, hepatic energy metabolism, nicotinamide adenine dinucleotide (NAD) salvage and transmethylation pathways and ultimately contributes to the alterations in hepatic lipogenesis and development of metabolic deficiencies.^6–8^ Clinical reports also demonstrate that chronic sleep loss (such as obstructive sleep apnea) is strongly associated with an increased risk of liver cirrhosis through the activation of the hypoxia inducible factor, nuclear factor-κB or unfolded protein response.^9–11^ By using molecular imaging and spectral analyses, we also demonstrate significant impairments in ionic homeostasis and phospholipid levels in hepatocytes following SD.^12,13^ It is indicated that enhanced cellular stress and intense oxidative damage in the liver may play vital roles in the pathogenesis of SD-related metabolic deficiencies.^12–14^ Considering the importance of the liver in controlling metabolic homeostasis in mammals, developing natural substances that are highly accessible to the liver and possess effective antioxidative activities may thus shed important light on advancing therapeutic strategies to prevent or counteract the SD-induced metabolic dysfunction prevailing in our societies nowadays.

Plasmon-activated water (PAW) is an innovative invention that possesses numerous advantages compared with conventional deionized water (DIW).^15^ The health benefits include the ability to scavenge free hydroxyl radicals and to effectively reduce the release of nitric oxide (NO) from lipopolysaccharide (LPS)-induced inflammatory cells.^16^ PAW consists of small water clusters with reduced hydrogen bonding and is responsible for high diffusion coefficients, which can be measured.^15^ By letting DIW flow through supported gold nanoparticles (AuNPs) under resonant illumination, effective hot electron transfer breaks the hydrogen bonds and thus makes PAW more active in various chemical and physical reactions.^15,16^ Moreover, a quantitative evaluation of the activated property-tunable PAW with reduced hydrogen bonds is successfully developed using deconvoluted Raman spectroscopy,^17^ promising innovative applications that are more flexible and reliable.

**Fig. 1:**
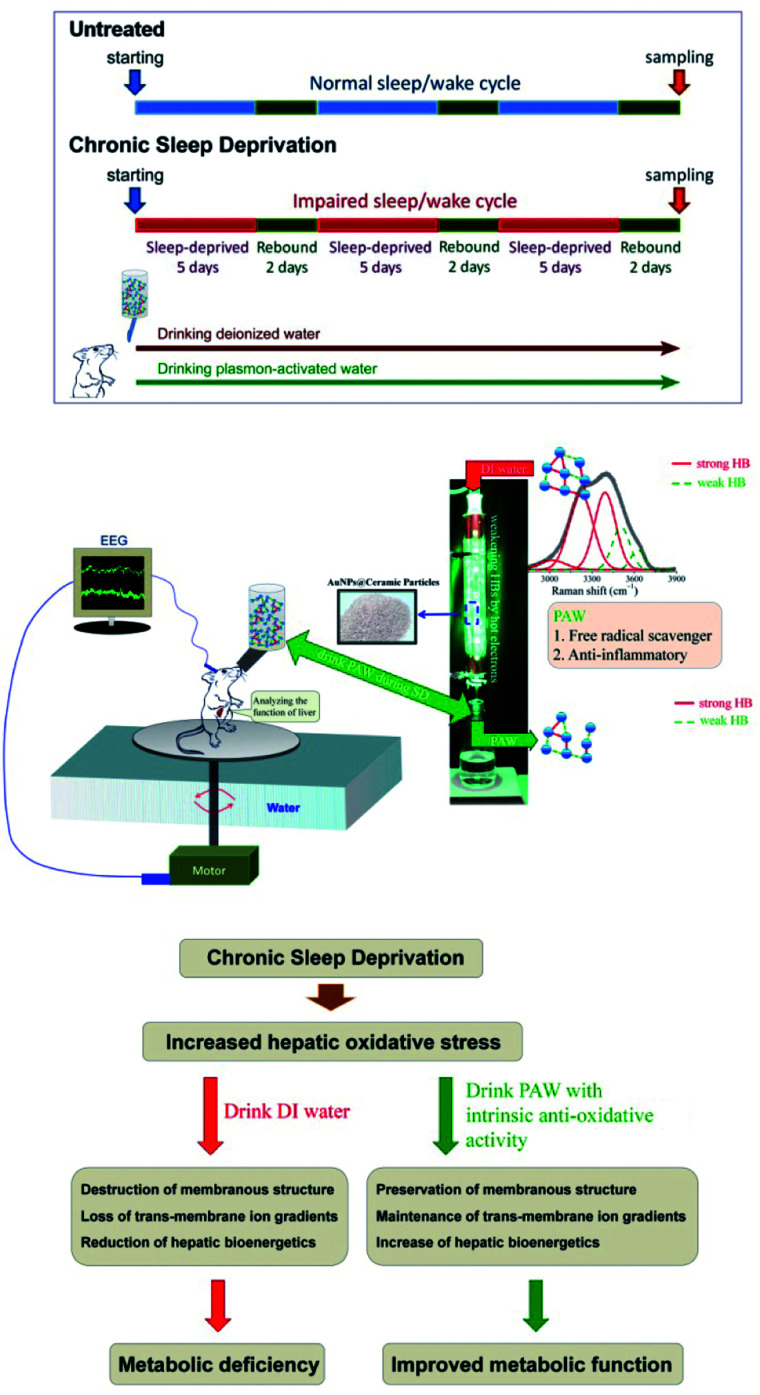
Schematic diagram showing the experimental design and the proposed mechanism(s) of the current study. CSD was processed for three cycles (with 5 days of total sleep deprivation followed by a 2 day break in each cycle). During the entire CSD period, rats were drank either DIW or PAW. Sleep deprivation was achieved by the disc-on-water method. When sleep onset was detected by the electroencephalography (EEG) machine in a sleep-deprived rat, the disc was slowly rotated at a moderate speed of 3.5 rpm by a computerized monitoring system, forcing the rat to remain awake and walk against the direction of the disc rotation to avoid being forced into the water. When a sleep-deprived rat was spontaneously awoken, the disc became stationary. During this process, the sleep-deprived rats drank either DIW or PAW. PAW was produced by treating DIW with excited AuNP-absorbed ceramic particles. PAW with weak hydrogen bonds (demonstrated by deconvoluting Raman spectra) exhibited efficient anti-oxidative and anti-inflammatory properties. The underlying mechanism(s) of the hepato-protective effects of PAW was proposed to be resulting from the intrinsic anti-oxidative activity of PAW that protects liver from CSD-induced oxidative damage.

A previous study indicated that single water molecules exhibit significant effects on slowing down oxidative reactions.^18^ A biochemical report also demonstrated that free hydrogen can act as a powerful antioxidant by selectively reducing cytotoxic oxygen radicals.^19^ These findings together with our previous report documenting the use of PAW to increase the hemodialysis efficiency and safety^16^ supported the therapeutic application of PAW as a potential (and natural) agent to reduce oxidative injury. As reported in the literature,^12,13^ enhanced oxidative stress certainly plays an important role in the pathogenesis of SD-induced liver dysfunction. Based on this viewpoint, exploring the potential effects of PAW on reducing hepatic oxidative stress following SD is worthy of a trial for its clinical use as a hepato-protective agent in a safer, more economical and more convenient way. Herein, we showed that drinking PAW daily by young rats suffering from SD is a healthy and natural strategy for counteracting SD-induced liver deficiencies. The animal model and experiment process are shown in Fig. 1. The SD process was repeated for three cycles. During chronic SD (CSD), rats drank DIW and PAW. The health benefits of PAW in scavenging free hydroxyl radicals and inhibiting inflammatory reactions were previously reported in our study.^13^ As intense oxidative damage in the liver is the major cause for the pathogenesis of SD-related metabolic dysfunction.^12–14^ We hypothesized that PAW with an intrinsic anti-oxidative activity would protect liver from intense oxidative damage during SD and consequently reduce SD-related metabolic deficiencies (Fig. 1). Because of the anti-oxidative properties of PAW, it functions like an anti-inflammatory medical drug, equivalent to what Esculentoside A does for patients with liver injury,^20^ but with fewer side effects.

## 2. Experimental

### 2.1. Preparation conditions of PAW

For preparing PAW, DIW (pH 7.23, *T* = 23.5 °C) was passed through a glass tube filled with Au NP-adsorbed ceramic particles under illumination. Then, PAW (pH 7.25, *T* = 23.3 °C) was collected in glass sample bottles for subsequent tests as soon as possible. For examining the purity of the prepared PAW further, inductively coupled plasma-mass spectrometer (ICP-MS) analyses indicated that the concentrations of the slightly dissolved metals in PAW were *ca.* 0.62, 43, 25, 23, 13, 4.5 and 0.41 ppb for Au, Na, K, Al, Mg, Ca and Fe, respectively. Excluding Au, the total equivalent molar concentration of these dissolved metals was equal to *ca.* 6.9 × 10^−6^ N. The measured value was *ca.* 2.4 × 10^−7^ N for DIW used as a reference. Moreover, the total equivalent molar concentrations of the slightly dissolved Au and other dissolved metals in PAW (light-free) were 0.57 ppb and 5.2 × 10^−6^ N, respectively.

### 2.2. Treatments of experimental animals and surgical procedures

Adult male Wistar rats (*n* = 36, weighing 200–250 g) obtained from the National Laboratory Animal Center (Taipei, Taiwan) were used in this study. Surgical procedures for electroencephalographic (EEG) and electromyographic (EMG) recordings were performed using our well-established methods as described previously.^19–21^ All rats under chloral hydrate anesthesia (0.4 mL/100 g) were restrained in a stereotaxic apparatus equipped with a heating pad. The cranium was exposed, and five stainless steel screws (Small Parts, Miami Lakes, FL, USA) were implanted in the skull to serve as dural electroencephalographic electrodes. The electrodes were soldered to connectors of a plug that was fixed to the skull with dental cement. For EMG recording, four stainless steel wires were inserted into the nuchal musculature. The experimental animals were then organized equally into three groups (Fig. 1). Rats in the first group (*n* = 12) were subjected to CSD for three cycles in which each cycle consisted of 5 days of total SD followed by a 2 day break. During the CSD period, DIW was provided daily for drinking (CSD + DIW group). The second group was subjected to CSD for the same period, but these rats were given PAW instead of DIW for drinking (CSD + PAW group). Animals in the third group (drinking DIW) were kept in plastic cages placed beside the experimental apparatus and served as normal untreated controls (untreated group). During the experimental period, all rats were exposed to an automatically regulated light–dark cycle of 12 : 12 h (lights on at 07 : 00–19 : 00 with the light intensity in the center of the room at about 300 lux and a wavelength of 253.7 nm) at a constant room temperature of 25 ± 1 °C. Animals were allowed *ad libitum* access to food and DIW or PAW. The procedures for caring and handling of all experimental animals were in accordance with the ethical standards laid down in the 1964 Declaration of Helsinki and its later amendments. All drug administration procedures were further approved by the Committee on the Care and Use of Laboratory Animals at the Taipei Medical University (LAC-2014-0062).

### 2.3. SD process and recordings

Total SD was achieved by the disc-on-water method as described in our previous studies.^21–23^ Briefly, the apparatus comprised two rectangular clear plastic chambers placed side by side. A single plastic disc, which served as a rat-carrying platform, was built into the lower quarter of each chamber. Beneath the disc, and extending into the chamber walls, was a rectangular tray filled with water to a depth of 5 cm. Before the experiment began, a rat which was to be sleep-deprived and its yoked control were placed in the SD apparatus for at least 3 days for environmental acclimation. SD depended on the rats' aversion to water as rats rarely enter water spontaneously. When sleep onset was detected in a sleep-deprived rat, the disc was slowly rotated at a moderate speed of 3.5 rpm by a computerized monitoring system, forcing both rats to remain awake and walk against the direction of the disc rotation to avoid being forced into the water. When a sleep-deprived rat was spontaneously awoken, the disc became stationary, and the yoked control rat had an opportunity to sleep. Electroencephalographic and electromyographic data were obtained using a Grass model 78 polygraph (Grass-Telefactor, West Warwick, RI, USA) and were relayed to a computer for digital recording.

### 2.4. Perfusion and tissue preparations

At the end of the experimental period (three cycles of SD with 7 days in each cycle), half of the animals in each experimental group were subjected to transcardial perfusion for advanced ionic imaging acquired from the time-of-flight secondary ion mass spectrometry (TOF-SIMS) analysis and quantitative histochemical/morphological studies. First, animals were deeply anesthetized with 7% chloral hydrate (0.4 mL kg^−1^) and then perfused with 0.9% saline followed by 300 mL of 2% paraformaldehyde and 2.5% glutaraldehyde in 0.1 M cacodylate buffer (PB) at pH 7.4. During perfusion, blood samples were immediately withdrawn from the left ventricle for the serum assay. After perfusion, the liver was removed and placed in the same fixative for 2 h. The tissue was then immersed in graded concentrations of sucrose buffer for cryoprotection at 4 °C. Serial 30 μm-thick sections of the liver were cut transversely with a cryostat (CM3050S, Leica Microsystems, Wetzlar, Germany) on the following day, and the sections were alternatively placed into three wells of a cell culture plate. The sections collected in the first well were processed for the TOF-SIMS analysis, and those in the last two wells were processed for scanning electron microscopy (SEM) and cytochrome c oxidase (COX) histochemistry, respectively.

### 2.5. COX histochemistry

A slightly modified method of Wong-Riley was used to demonstrate the COX reactivity in our present study.^24^ The reaction medium contained 0.03% cytochrome c, 0.05% 3,3′-diaminobenzidine, and 0.02% catalase (all from Sigma, St. Louis, MO, USA) in 0.1 M PB at pH 7.4. Sections collected from the third well were incubated in this medium at 4 °C in dark overnight. After incubation, the sections were rinsed for 20 min in 0.1 M PB followed by a rinse with distilled water to terminate the reaction. All the reacted sections were rapidly dehydrated through a graded series of alcohol, cleared with xylene, and covered with a coverslip and Permount.

### 2.6. Measurement of liver lipid peroxidation

As malondialdehyde (MDA) is the most abundant product resulting from lipid peroxidation, measurement of MDA is extensively used as an index of oxidative stress.^25^ MDA levels were measured by the method described by Silva *et al.*^26^ The remaining rats from all the experimental groups were killed by deep anesthetization with 7% chloral hydrate, and their livers were immediately excised. Then, the livers were homogenized in ice-cold 0.1 M PB. The homogenates were centrifuged at 4000 rpm for 10 min at 4 °C, and the supernatants were collected to detect MDA by measuring the fluorescence product formed from the reaction of this aldehyde with thiobarbituric acid. The results were determined spectrophotometrically at 532 nm to obtain the MDA levels using tetraethoxypropane as a standard and were expressed in nanomoles per milligram.

### 2.7. Western blot analysis of a stress protein (HSP-27)

For the Western blot analysis, livers removed from decapitated animals were frozen in liquid nitrogen and then homogenized with 100 μL lysis buffer on ice using a grinder. The Western blot procedure was processed by methods described previously.^27^ Briefly, 10 μg of solubilized proteins was separated by the sodium dodecylsulfate polyacrylamide gel electrophoresis (SDS-PAGE; 12%) and was electroblotted onto a polyvinylidene difluoride (PVDF) membrane (Bio-Rad Laboratories, Hercules, CA, USA). The membranes were blocked with 5% non-fat dry milk and sequentially probed with antibodies against β-actin (1 : 5000) and HSP-27 (1 : 1000). Following this, the PVDF sheets were incubated with a horseradish peroxidase-conjugated secondary antibody at a dilution of 1 : 5000 for 1 h at room temperature. The immunoreaction was visualized with an enhanced chemiluminescence (ECL) solution (5 min) followed by film exposure for 2 min. OD was quantified with the computer-assisted software (Science Lab 2003, Fuji, Japan). Densitometric results were normalized against β-actin and were presented as the mean ± standard deviation.

### 2.8. Measurement of antioxidant enzyme activities

The activity of the total superoxide dismutase (SOD) was according to that described by McCord and Fridovich^28^ and was based on the production of superoxide radicals during the conversion of xanthine to uric acid by xanthine oxidase and the inhibition of the reduction of cytochrome c. One unit of SOD activity was defined as the amount of SOD that produced 50% inhibition of cytochrome c reduction. The catalase activity was assayed using the method described by Clairbone.^29^ One mL of the reaction mixtures consisted of 50 mM potassium phosphate, 19 mM H_2_O_2_, and a sample. The reaction was initiated by the addition of H_2_O_2_, and absorbance changes were measured at 240 nm. The glutathione peroxidase activity was determined by measuring the conversion of NADPH to NADP in the presence of reduced glutathione and H_2_O_2_ spectrophotometrically at 340 nm.^30^

### 2.9. Biochemical determination of serum levels of liver function and metabolic markers

Serum levels of liver function [*i.e.* alkaline phosphatase (ALP), glutamic oxaloacetic transaminase (GOT), glutamic pyruvic transaminase (GPT), total protein and albumin] and some metabolic markers [*e.g.* glucose, triglyceride, insulin, high-density lipoprotein (HDL), and low-density lipoprotein (LDL)] were determined using commercially available kits. All analyses were performed according to the manufacturer's protocols.

### 2.10. Analysis of cell viability

To evaluate the cytotoxicity of PAW, a MTT [3-(4,5-dimethylthiazol-2-yl)-2,5-diphenyl-tetrazolium bromide] assay was performed to determine the cell viability. Briefly, the rat liver cells (Clone-9) were seeded at a density of 8 × 10^4^ cells per well in a 24-well plate for 16 h. Following that, cells were incubated in DIW DMEM medium or PAW DMEM medium for 24 h. After the exposure period, the medium was removed, and this was followed by washing the cells with PBS. Then the medium was changed, and the cells were incubated with MTT solution (0.5 mg mL^−1^) for 4 h. After removing the medium, the formazan was solubilized in isopropanol and measured spectrophotometrically at 563 nm. The percentage of viable cells was estimated by comparing with that of the untreated control cells.

### 2.11. Cell proliferation assay

A 3-(4,5-dimethylthiazol-2-yl)-2,5-diphenyl tetrazolium bromide colorimetric assay was developed to monitor the mammalian cell proliferation *in vitro*. The Clone-9 cells were plated at an initial density of 1 × 10^4^ cells per well in DMEM containing 10% FCS. After overnight attachment, cells were incubated in DMEM medium or PAW DMEM medium for 1 to 5 days. At the end of each final 4 hours, MTT solution (0.5 mg mL^−1^) was added to each well. The formazan was solubilized in isopropanol and measured spectrophotometrically at 563 nm.

### 2.12. Detecting cell migration activity with gelatin zymography

The matrix metalloproteinase-2 (MMP-2) activity was evaluated using gelatin zymography. The Clone-9 cells were incubated in DMEM medium or PAW DMEM medium for 24 h after plating 8 × 10^4^ cells in 24-well plates for 16 h. The harvested culture medium containing 10 g of total protein was subjected to SDS-PAGE containing 0.1% gelatin. After electrophoresis, the gel was washed with a washing buffer (2.5% Triton X100) to remove SDS and then incubated at 37 °C in a reaction buffer containing 40 mM Tris–HCl, 10 mM CaCl_2_, and 0.01% NaN_3_. The proteolytic activity of MMP-2 was obtained by staining with Coomassie Brilliant Blue R-250. The intensity of unstained bands on the blue background was measured by spot density measurement using a densitometer (AlphaImager 2000, Alpha Innotech Corp., San Leandro, CA).

## 3. Results

### 3.1. PAW reduces hepatic oxidative stress

**Fig. 2:**
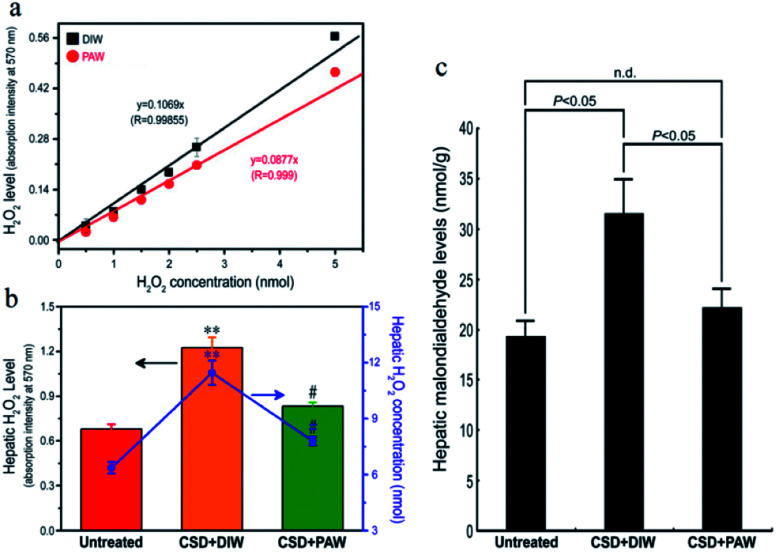
Line chart (a) and histograms ((b) and (c)) showed the anti-oxidative effects of PAW on liver as determined by both (a) *in vitro* and ((b) and (c)) *in vivo* assessments. Please note that PAW significantly decreased the H_2_O_2_ level of rat liver Clone-9 cells (a). Also note that drinking PAW successfully reduced the CSD-induced hepatic oxidative stress (c) by effectively suppressing hepatic H_2_O_2_ levels (b). ^#^*p* < 0.05 when compared with that of CSD + DIW group, and **p < 0.01 when compared with that of untreated group.

As designated in Fig. 2, CSD (with consumption of DIW) produced excessive amounts of H_2_O_2_ in the livers of the animals and led to intense oxidative stress. However, in animals drinking PAW instead of DIW during the entire CSD period, both the hepatic H_2_O_2_ and malondialdehyde (MDA, a reliable marker of oxidative stress^23^) levels were drastically reduced to nearly normal untreated values (Fig. 2b and c). The positive effects of PAW on reducing oxidative stress may arise from the scavenging activity of PAW on H_2_O_2_, since the quantity of H_2_O_2_ prepared by the standard process with PAW was significantly lower than that prepared with DIW (Fig. 2a).

### 3.2. PAW preserves hepatic ion homeostasis

**Fig. 3:**
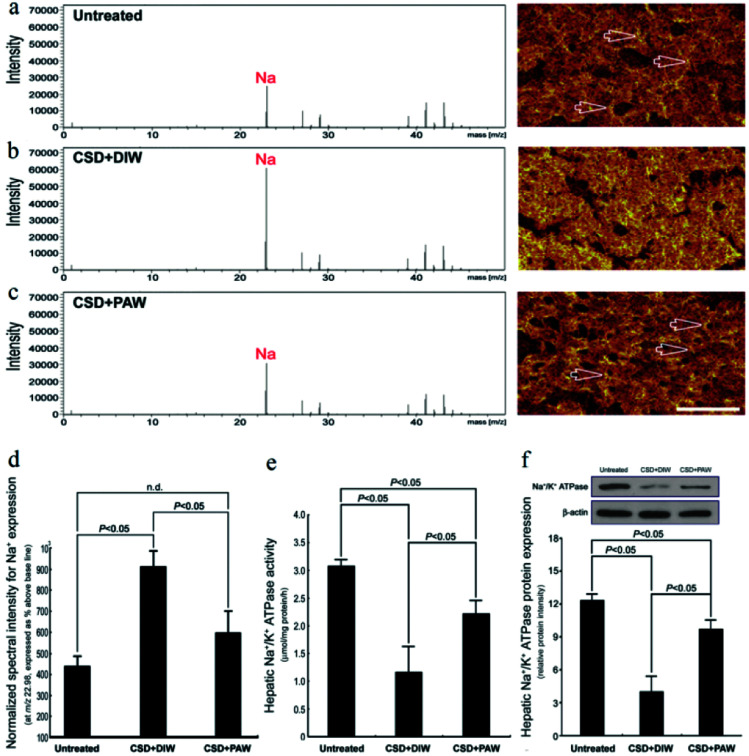
Effects of PAW on preserving Na^+^/K^+^ ATPase function and restoring the *trans*-membrane ionic gradient following CSD injury. Positive spectra/ionic images showed that in the normal untreated rats, most of the Na^+^ signals were localized to the extracellular portion of the hepatocytes [arrows in (a)]. Following CSD, strong Na^+^ signals were detected in the cytoplasmic portion of the hepatocyte (b), indicating the impairment of *trans*-membrane ionic regulation (b). However, in animals supplied with PAW everyday during the entire CSD period, the distribution pattern of Na^+^ was much similar to that of normal untreated ones in which the majority of Na^+^ were localized to the extracellular sinusoid space [arrows in (c)]. The corresponding data of the normalized spectral intensities for a–c (d). Biochemical data coincided well with ionic imaging findings in which PAW effectively preserved hepatic Na^+^/K^+^ ATPase expression (f) and improved Na^+^/K^+^ ATPase activity (e).

TOF-SIMS revealed that in the normal untreated rats, most of the Na^+^ signals were distributed in the extracellular portion (*i.e.*, hepatic sinusoids) of hepatocytes (arrows in Fig. 3a). However, following CSD, strong Na^+^ signals were accumulated in the intracellular portion of hepatocytes (Fig. 3b). The enhanced cytoplasmic Na^+^ expression indicated an intracellular Na^+^ overload, which could lead to ionic dyshomeostasis and consequently contribute to the development of cytosolic acidification and metabolic deficiencies.^29^ Nevertheless, in animals subjected to CSD and provided with PAW, the hepatic Na^+^ expression successfully returned to nearly normal levels in which the majority of Na^+^ was distributed in the extracellular portion of hepatocytes (arrows in Fig. 3c). The findings of ionic images (Fig. 3a–c) and the data of the normalized spectral intensity (Fig. 3d) coincided well with the results of the hepatic Na^+^/K^+^ ATPase activity and protein assay, wherein the impairment (or recovery) of ionic homeostasis paralleled the reduction (or increment) in Na^+^/K^+^ ATPase activity (Fig. 3e and f). Based on these findings, it was suggested that daily drinking of PAW during CSD can effectively preserve Na^+^/K^+^ ATPase function and successfully restore the ionic gradient to a normal stage.

### 3.3. PAW protects plasma membrane integrity

**Fig. 4:**
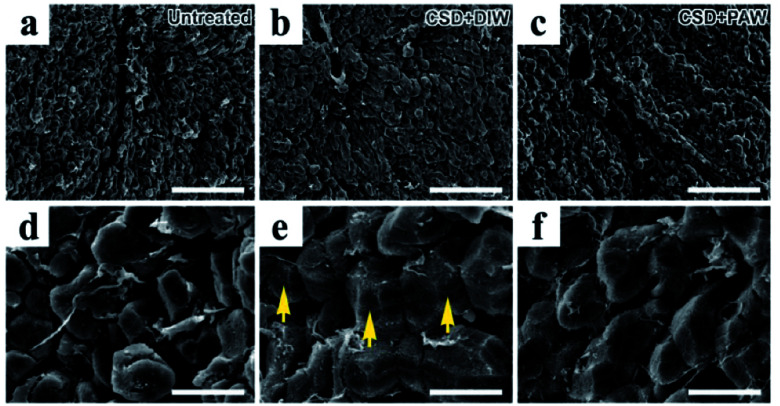
Scanning electron microscopy showed morphological changes in oxidative stress in hepatocytes following CSD injury. The detrimental effects of oxidative stress were evidently demonstrated by the significant lipid peroxidation of plasma membranes [arrows in image (e)]. Note that daily drinking of PAW (when compared with drinking of DIW) during CSD successfully preserved the integrity of hepatocytic membranous structures from oxidative damage ((c) and (f)). Scale bar = 120 μm in ((a)–(c)); scale bar = 20 μm in ((d)–(f)).

Morphological changes in hepatocytes were examined by scanning electron microscopy (SEM). In normal untreated rats, nearly all hepatocytes were found to have intact plasma membrane structure (Fig. 4a and d). Following CSD, significant defects in the membranous structure were observed in hepatocytes, in which a variety of surface damages (arrows in Fig. 4e) were identified in the plasma membranes. The plasma membranes of hepatocytes are mainly composed of phospholipids. Impairment of membrane structures after CSD might manifest morphological features of lipid peroxidation, a detrimental effect of oxidative damage noticeably demonstrated in the current study (Fig. 2c). However, in animals drinking PAW during the entire period of CSD, nearly no obvious impairment was identified in the membranous structures of the hepatocytes (Fig. 4c and f). This result indicated that PAW can exert a protective effect on hepatocytes following CSD by successfully preserving the integrity of hepatocytes from CSD-induced oxidative injury.

### 3.4. PAW improves hepatic bioenergetics

**Fig. 5:**
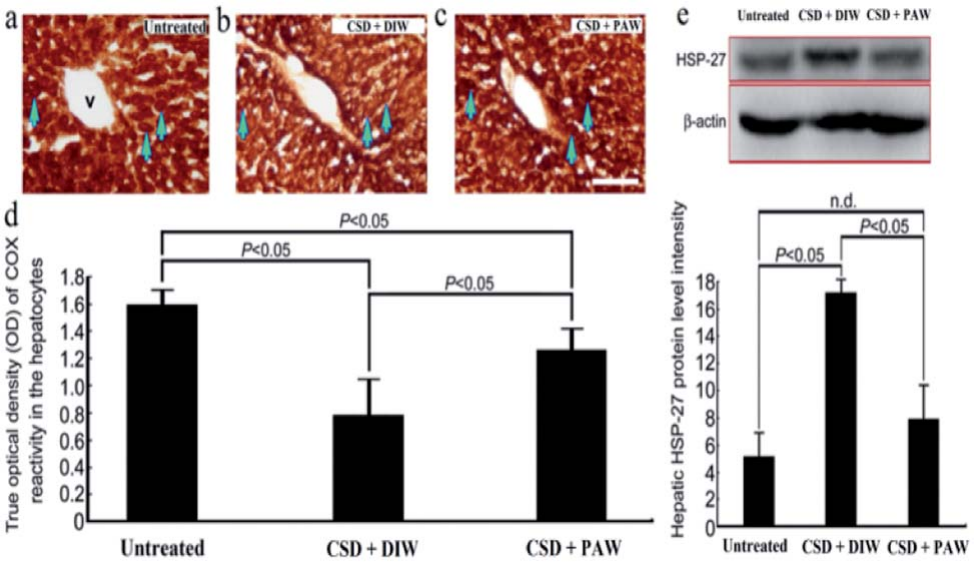
Photomicrographs ((a)–(c)) and histograms ((d) and (e)) showed the hepato-protective effects of PAW on preserving the cellular bioenergetics [as determined by cytochrome oxidase (COX) reaction] ((a)–(d)), and reducing the stress level [as expressed by heat shock protein-27 (HSP-27) immunoblots] (e) following CSD injury. Note that CSD significantly depressed the cellular bioenergetics and increased hepatic stress level as shown by decreased COX staining [arrows in (b)] and enhanced HSP-27 activity (e). However, in case of animals drinking PAW during the entire CSD period, effective increment in COX staining [arrows in (c)] combined with reduced HSP-27 expression (e) was detected in the hepatic tissues. V: central vein. Scale bar = 100 μm in ((a)–(c)).

**Fig. 6:**
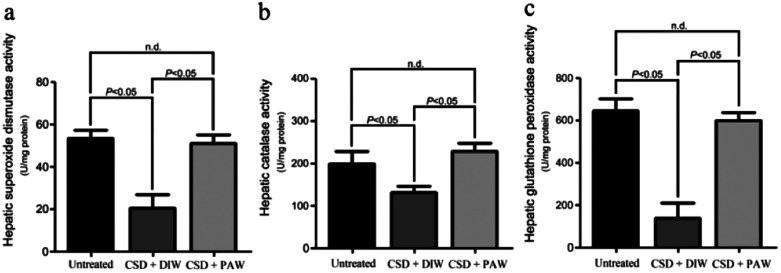
Histograms showed the hepato-protective effects of PAW on increasing the activities of anti-oxidant enzymes following CSD injury. Please note that daily drinking of PAW during the entire CSD period would significantly preserve the hepatic superoxide dismutase (a), catalase (b) as well as glutathione peroxidase (c) activities.

Hepatic bioenergetics were determined by simultaneously examining the cytochrome oxidase [(COX), a reliable cellular energetic marker^22^] staining, heat shock protein 27 (HSP-27, a cellular stress marker^25^) immunoblot expression, and several antioxidant enzyme [*e.g.* superoxide dismutase (SOD), calatase, and glutathione peroxidase (GPx)] activities. Histochemical results indicated that numerous hepatocytes with strong COX staining were observed in the livers of the normal untreated rats (arrows in Fig. 5a). The enhanced COX expression well paralleled the lower level of HSP-27 activity (Fig. 5e). However, following CSD, significant up-regulation of HSP-27 expression (Fig. 5e), decreased COX staining (arrows in Fig. 5b) together with reduced SOD, catalase, and GPx activities (Fig. 6) were detected in the hepatocytes. Nevertheless, in rats subjected to CSD and provided with PAW, the hepatic bioenergetic level was successfully improved as revealed by enhancing the energetic marker (arrows in Fig. 5c), increasing the antioxidant enzyme activities (Fig. 6) as well asreducing the level of stress protein (Fig. 5e). These findings suggested that PAW could improve the hepatic bioenergetics and exert a powerful hepato-protective effect on those subjects experiencing severe SD-induced liver injury.

### 3.5. PAW restores liver and metabolic functions

**Fig. 7:**
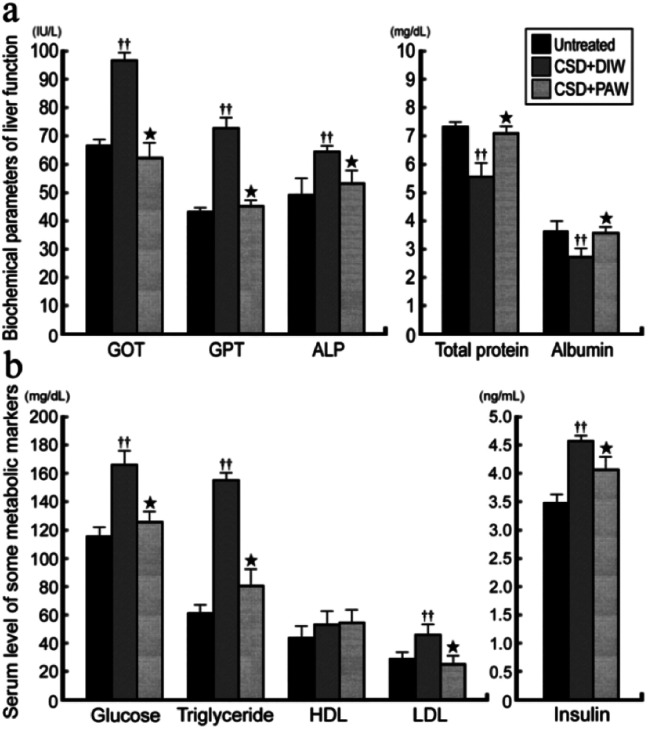
Histograms showing the serum level of biochemical markers related to liver (a) and metabolic (b) functions. Note that CSD contributes to severe liver and metabolic deficiencies. However, drinking of PAW successfully exerts beneficial effects on liver and metabolic function in which almost all biochemical markers were noticeably returned to nearly normal values.

ALP, GOT, GPT, and albumin/total protein were used to reveal the liver function of normal untreated and CSD rats with or without PAW treatment. The results indicated that in normal untreated rats, concentrations of GOT, GPT, and ALP were all within normal ranges (Fig. 7a). Following CSD, the impairment of liver and metabolic functions was clearly demonstrated by the enhanced GOT, GPT, and ALP levels together with the incidences of hyperglycemia, hypertriglyceridemia, hyperlipidemia, and hyperinsulinemia (Fig. 7b). However, in rats subjected to CSD and provided everyday with PAW, both the liver and metabolic functions were gradually returned to nearly normal values (Fig. 7a and b).

## 4. Discussion

This study provides the first functional anatomical evidence of health benefits, particularly in hepato-protection, resulting from the daily drinking of PAW. The benefits include successful reduction of hepatic oxidative stress (Fig. 2), maintenance of hepatocytic *trans*-membrane ion gradients (Fig. 3), preservation of hepatocytic membrane structures (Fig. 4), increase in hepatic bioenergetics (Fig. 5 and 6), and ultimate contributions to improved liver and metabolic functions following CSD (Fig. 7). The beneficial effects of PAW may be attributed to its significant anti-oxidative abilities to scavenge free radicals and effectively reduce inflammation in cells.^15,16^ However, the retention of PAW with special properties in rats is of concern. In our previous reports, it has been demonstrated that PAW has weaker hydrogen bonded interaction than does DIW within water molecules due to the destruction by hot electrons from the decay of excited gold nanoparticles (AuNPs).^15,32^ Because of the weak interaction and hot electrons doping, PAW exhibits low specific heat, high vapor pressure, high osmosis and negative charge. Especially, PAW shows the properties of anti-inflammation and free radical scavenging. Moreover, such an anti-inflammatory experiment has been performed using PAW 264.7 cells. This means that PAW can pass through cell membranes and can retain its intrinsic property to inhibit inflammation. The stability of PAW has also been investigated. For the pure PAW without other additives, the functions of PAW are decayed with time and reverted to the normal state within 5–7 days.^15^ In the presence of H_2_SO_4_ (0.5 to 1 M), the stability of PAW can extend for more than 7 days.^17,33^ Besides, PAW can also retain its properties in alkaline solution.^34^ This means that PAW is stable in neutral, acidic and alkaline environments such as the stomach, liver and gut, respectively. This is just the case in our current study. PAW effectively suppresses the damage of hepatocytes caused from the reactive oxygen species (ROS) of H_2_O_2_ (Fig. 2). Literatures report that water is favorable for the catalytic effect on radical–radical (H_2_O_2_–OH) reactions due to the ability of water to form a stable complex (HO_2_·H_2_O) with HO_2_ radical through hydrogen bonding,^35,36^ in which the binding energy of HO_2_·H_2_O is 30.9 kJ mol^−1^, which is higher than 23 kJ mol^−1^ of hydrogen bond within water molecules. This stable complex can potentially modify the reactivity of HO_2_. Meanwhile, the proton-transferred hydrogen-bonded complexes from HO_2_·H_2_O to O_2_·H_3_O^+^ might be formed.^37^ A previous report demonstrates that PAW provides more amount of activity sites for forming hydrogen bonds with other species compared with DIW. According to eqn (1) to eqn (3),^35,36^ the amount of hydrogen-bonded H_2_O_2_·H_2_O, H_2_O·OH and HO_2_·H_2_O in PAW would be higher than those in DIW due to the highly active sites of PAW, which means that more amount of H_2_O_2_ is consumed in PAW, which could be a H_2_O_2_ scavenger.

1 H_2_O_2_·H_2_O + OH → HO_2_ + 2H_2_O

2 H_2_O_2_ + H_2_O·OH → HO_2_ + 2H_2_O

3 HO_2_·H_2_O → O_2_·H_3_O^+^

The evidence of scavenging H_2_O_2_ by PAW is obtained using a H_2_O_2_ assay kit. It is observed that the quantity of H_2_O_2_ prepared by the standard process based on DIW is proportional to the absorption intensity at 570 nm with a linear slope of 0.1069 (Fig. 2a). The slope decreases to 0.0877 as DIW is replaced by PAW. The positive effects of PAW on reducing the oxidative stress may arise from the scavenging activity of PAW on H_2_O_2_, since the quantity of H_2_O_2_ prepared by the standard process with PAW is significantly lower than that prepared with DIW. This result parallels well with the findings of the *in vivo* study in which both the level of H_2_O_2_ (Fig. 2b) and hepatic oxidative stress (Fig. 2c) following CSD were much lower in PAW group when compared with those in DIW group. Although it is difficult to directly trace PAW in animals because the Raman spectrum, vapor pressure, heat capacity and boiling point, which are used to characterize PAW, are easily influenced by the presence of species (such as salts and proteins) existing in the liver, the effects of PAW on depressing the hepatic H_2_O_2_ level and oxidative stress highly support the presence of PAW in liver tissues. In addition, another indirect evidence for the presence of PAW in the liver is further provided by detecting the concentration of gold. The result of ICP-MS indicates that nearly no gold signal was detected in the liver of the DIW group. However, in animals drinking PAW during the entire CSD period, about 0.51 ng of gold per gram of liver tissue is detected, suggesting that PAW is actually present in the liver. Moreover, the possible cytotoxicity of PAW has further been excluded by our *in vitro* studies in which the cell viability of liver cells (Clone-9)^38,39^ cultured in PAW is comparable to that cultured in DIW (Fig. S1a†). PAW also promotes the proliferative activity of liver cells (Fig. S1b†) without changing the cellular migration capacity (Fig. S1c†). With regard to these findings, it is suggested that PAW can serve as a natural and safe hepato-protective agent through directly scavenging H_2_O_2_ and reducing the oxidative stress induced by chronic sleep deprivation injury.

On the other hand, abnormalities in glucose and lipid profiles following CSD may also disrupt the redox status and contribute to the formation of oxidative stress.^40^ Overproduction of ROS can destroy lipid structures, which subsequently causes lipid peroxidation damage.^41,42^ Impairments of the plasma membranes due to lipid peroxidation disturbs Na^+^/K^+^ ATPase, an important membrane-bound enzyme responsible for controlling osmotic regulation and ionic homeostasis. Inactivation of Na^+^/K^+^ ATPase lowers the membrane barrier for the movement of Na^+^ such that it can more easily enter the cytoplasm.^43^ This is just the case in our present study, in which excessive Na^+^ influx is detected in hepatocytes as a result of depressed Na^+^/K^+^ ATPase (Fig. 3b,e and f) activity. Enhanced cytosolic Na^+^ results in an osmotic overload and cell swelling that ultimately leads to membrane rupture (Fig. 4e) and hepatocellular injury (Fig. 5e).^44^ In addition, unbalanced intracellular Na^+^ accumulation can also impair electrophysiological homeostasis and interrupt the mitochondrial electron transport chain, thereby giving rise to the development of bioenergetic deficiencies.^45^ Our current findings thus coincide well with this viewpoint in which we further detected reduced COX expression (the terminal enzyme of the mitochondrial electron transport chain) in hepatocytes following CSD (Fig. 5b and d). These biochemical changes may therefore serve as the underlying mechanisms for the pathogenesis of CSD-induced metabolic deficiencies (Fig. 7). However, in animals that daily drank PAW during the entire CSD period, all of the above parameters successfully return to nearly normal levels (Fig. 3c–f, 4c and f, 5c and e, and 6). The hepato-protective effects of PAW are functionally demonstrated by the reduced GOT and GPT values and recovery of total protein and albumin levels (Fig. 7). An extensive increase in hepato-specific enzymes following CSD indicates hepatocellular damage, and reductions in these enzymes by PAW strongly indicates that the consumption of PAW can exert a powerful effect on improving the liver function and preserving the metabolic activity following CSD (Fig. 7).

In this study, SD was achieved by the disc-on-water method, which may exert some stress to the animals resulting from forced movement. In this regard, we designed a yoked control that was housed in the same disc but had the opportunity to sleep when the disc was stationary due to the experimental animals being spontaneously awakened. This strategy effectively eliminated the potential confound induced by locomotion-related stress, since both experimental and control rats received the same amount of movement. However, it is noteworthy that in this apparatus, the rats spent most of their time with their tails in the water. As tail of a rat is one of the thermoregulatory measurements, the potential impact of thermo-disruption on the metabolic function following SD should not be overlooked. Nevertheless, while not much is known about the potential influence of thermo-disruption on the metabolic function in this study, it is still reliable to assume that PAW is effective since drinking only PAW (*i.e.* one dosage from oral intake without any extra administration) significantly reveals powerful hepato-protective function following chronic sleep deprivation injury.

## 5. Conclusions

Based on these findings, it was concluded that PAW can effectively protect the liver from CSD-induced oxidative injury. The primary mechanism(s) by which PAW bearing intrinsic anti-oxidative activity protects the liver from intense oxidative damage as well as preserves hepatic bioenergetics and reduces hepatic stress during CSD is proposed, which can reduce pathogenesis of CSD-related metabolic deficiencies. The anti-oxidative PAW functions like an anti-inflammatory medical drug for patients with liver injury, but with fewer side effects. Considering that CSD is an extremely common phenomenon in the present-time with deleterious effects on metabolic function, daily drinking of PAW may thus have great potential for its clinical use to obtain a novel strategy to protect the liver and metabolic function following CSD in an easier, healthier, more natural, and more convenient way.

## Conflicts of interest

There are no conflicts to declare.

## Supplementary Material

RA-008-C7RA13559A-s001
