# Supplementary material for: Plasmon-activated water effectively relieves hepatic oxidative damage resulting from chronic sleep deprivation
Source: RSC Adv. 2018 Mar 5;8(18):9618–26. doi: 10.1039/c7ra13559a (PMC9078654; doi:10.1039/c7ra13559a)
Supplement: RA-008-C7RA13559A-s001 [file RA-008-C7RA13559A-s001.pdf]

Electronic Supplementary Material (ESI) for RSC Advances.
This journal is © The Royal Society of Chemistry 2018

Supplementary Information

Plasmon-activated water effectively relieves hepatic oxidative damage resulted from chronic sleep deprivation

Hsiao-Chien Chen,‡[a] Chung-Yi Cheng,‡[b] Li-You Chen,‡[c] Chun-Chao Chang,[d] Chih-Ping Yang,[a] Fu-Der Mai,[a] Wen-Chieh Liao,[c] Hung-Ming Chang*[e] and Yu-Chuan Liu*[a]

[a] Department of Biochemistry and Molecular Cell Biology, School of Medicine, College of Medicine, Taipei Medical University, 250 Wuxing St., Taipei 11031, Taiwan.
[b] Division of Nephrology, Department of Internal Medicine, Taipei Medical University Wan Fang Hospital, 111 Hsing-Long Rd., Sec. 3, Taipei 116, Taiwan.
[c] Department of Anatomy, School of Medicine, College of Medicine, Chung Shan Medical University, 110 Sec. 1, Jianguo N. Rd., Taichung 40201, Taiwan.
[d] Division of Gastroenterology and Hepatology, Department of Internal Medicine, School of Medicine, College of Medicine, Taipei Medical University and Taipei Medical University Hospital, 250 and 252 Wuxing St., Taipei 11031, Taiwan.
[e] Department of Anatomy and Cell Biology, School of Medicine, College of Medicine, Taipei Medical University, 250 Wuxing St., Taipei 11031, Taiwan.

‡ These authors contributed equally to this work.
* Correspondence and requests for materials should be addressed to H.-M. Chang (E-mail: taiwanose@tmu.edu.tw) and Y.-C. Liu, (E-mail: liuyc@tmu.edu.tw).

Supplementary Materials and Methods

1. Chemicals and Materials

Electrolytes of NaCl (99+%) and KCl (99+%) were purchased from Sigma-Aldrich Organics. H_2O_2 was purchased from Acros Organics. The forty mesh-screened ceramic particles (Molar compositions: 92 % SiO_2, 3.0 % Na_2O and K_2O, 2.0 % Fe_2O_3, 1.5 % Al_2O_3, 0.5 % CaO, 0.5 % MgO, and other rare metal oxides) for filtering through drink water were purchased from Chyuan-Bang enterprise Co., Ltd., Taiwan. Commercial chitosan (Ch) powders with a degree of deacetylation of 0.82 were purchased from First Chemical Works, Taiwan. All of the solutions were prepared using deionized 18.2 MΩ cm water (DIW) provided from a Milli-Q system.

2. Preparation of gold nanoparticles (AuNPs)

The AuNPs in an aqueous solution obtained from an Au sheet (purity of 0.9999) by using electrochemical and thermal reduction methods.[46] Typically, the Au electrode was cycled in a deoxygenated aqueous solution of 40 mL containing 0.1 M NaCl and 1 g L^{-1} Ch from -0.28 to +1.22 V vs Ag/AgCl at 500 mV s^{-1} for 200 scans under slight stirring. The durations at the cathodic and anodic vertices are 10 and 5 s, respectively. Immediately, without changing the electrolytes, the solution was heated from room temperature to boiling at a heating rate of 6°C min^{-1} in air. After cooling the clear AuNPs-containing solution was separated from the settlement of Ch. Then the AuNPs-containing solution was placed in an ultrasonic bath for 30 min and was further centrifugalized at 3600 rpm for 2 min to remove Ch for preparing pure AuNPs in solution. The properties of the prepared AuNPs were shown in our previous report.[46] Also, the used chitosan in the preparation of AuNPs is a well-known biocompatible agent.

3. Preparation of ceramic particles-supported AuNPs and PAW

The rinsed ceramic particles were immersed in a solution containing 30 ppm AuNPs for 1 day. Then the AuNPs-adsorbed ceramic particles were rinsed throughout with DIW, and finally dried in an oven at 100°C for 1 day. Subsequently, the prepared AuNPs-adsorbed ceramic particles were loaded in a valve-equipped glass tube (I.D.: 30 mm, L: 300 mm). Before treating water, the AuNPs-adsorbed ceramic particles in the glass tube were rinsed with DIW for several cycles until the pH value is constant (ca. pH 7.23 and water temp. at ca. 23.5 °C). Based on the prepared ceramic particles-supported AuNPs, PAW was prepared following the method shown before.[15] The detailed PAW realization and properties were previously reported in the literature.[15-17]

4. TOF-SIMS analysis for Na^+ intensity and ionic imaging

The *in vivo* Na^+ expression and ionic imaging of Na^+ were assessed with the use of the TOF-SIMS analysis. The TOF-SIMS analysis was carried out on a TOF-SIMS IV instrument (ION-TOF, Münster, Germany) as described in our previous studies.[7,8] A gallium (Ga^+) ion gun operated at 25 kV was used as the primary ion source (1 pA pulse current). The Ga^+ primary ion beam was scanned over an area of 100 μm^2 which included 128 × 128 pixels. Positive secondary ions flying through a reflectron mass spectrometer were detected with a micro-channel plate assembly operating at 10 kV post-acceleration. Mass calibration of the ion spectrum was achieved using a set of mass peaks of *m/z* 15 (CH_3^+), 41 ($C_3H_5^+$), and 69 (Ga^+), and paraformaldehyde molecules since this element was the major component in the tissue matrix following vascular fixation[7]. Ions related to *m/z* 23 were used to identify and evaluate the ionic image of Na^+ expression.

5. Computerized image analysis for hepatic bioenergetics

The general approach for the computerized image analysis was similar to those in our previous study.[47] The staining intensity of COX was quantified with a computer-based image analysis system (MGDS) along with Image-Pro Plus software (Media Cybernetics, Silver Spring, MD, USA). A digital camera mounted on a Zeiss microscope (Axioplane 2, Carl Zeiss MicroImaging, Hamburg, Germany) imaged sections at 50× magnification in a bright field and displayed them on a high-resolution monitor. All densitometric readings taken from hepatocytes in each section were then combined and averaged to obtain the total OD (TOD) of each section. The background staining (BOD) of each section was measured by averaging 5 random rectangles (each with an area of 150 μm^2) of the vascular region within the central vein. The true OD for each section was then expressed by subtracting the BOD from the TOD, so that each measurement was made in an unbiased way to correct for the background. All images were captured on the same day by the same experimenter to maintain uniform settings adjusted at the beginning of capture. As the actual amount of reaction product deposited in a tissue section as a result of enzyme activity is influenced by a variety of factors, all parameters were carefully controlled following recommended procedures for gray level adjustment, histogram stretching, and minimal OD.[48]

6. Scanning electron microscopy (SEM) observations

For SEM observations, samples collected in the second well were mounted on aluminum sample holders and sputter-coated with a thin layer of gold-platinum. The micro-architecture features of hepatocytes were observed using SEM (Hitachi SU3500, Japan) in the secondary electron (SE) mode at an accelerating voltage of 20 kV.

7. Statistical analysis

For the TOF-SIMS analysis, the spectral intensity detected from each section was normalized to the ion intensity of paraformaldehyde (which served as a baseline = 100%) and was

expressed as a percentage above the baseline. All of the normalized spectra collected from each animal were then averaged to obtain representative data for that animal. The representative data acquired from animals belonging to the same experimental group were further averaged to yield the mean value for that corresponding group. Comparisons among mean values obtained from different experimental groups and other data acquired from spectrometric, biochemical, morphological, and neurochemical methods were subjected to the Kolmogorov-Smirnov test to analyze the pattern of normality. Those qualified were subsequently processed by a one-way analysis of variance (ANOVA) followed by the Bonferroni *post hoc* test. Statistical difference was considered substantial at $p < 0.05$.

8. *In vitro* and *in vivo* analyses of hepatic hydrogen peroxide (H_2O_2) levels

The anti-oxidative effects of PAW in both *in vitro* and *in vivo* assessments were evaluated from hepatic H_2O_2 levels based on its concentrations. A H_2O_2 standard curve was produced using an H_2O_2 assay kit (BioVision, Milpitas, CA, USA) and measuring the corresponding optical density (OD) at 570 nm. For this measurement, DIW, which was used to dilute the H_2O_2, was replaced by PAW to evaluate its ability to scavenge H_2O_2. *In vivo* experiments of the anti-oxidative effects of DIW or PAW were performed by measuring the corresponding H_2O_2 concentrations in the liver following CSD injury. For this measurement, 0.3 g of frozen liver collected from both normal untreated and CSD rats were rapidly triturated, and then 500 μL of DIW or PAW was added to extract the H_2O_2. After high-speed (5000 rpm) centrifugation, 50 μL of the supernatant was taken out and mixed with 50 μL of the H_2O_2 assay kit reagent. H_2O_2 concentrations were obtained by measuring the corresponding OD at 570 nm. The detection limit can be as low as 2 pmol per assay (or 40 nM concentration) of H_2O_2 in the sensitive fluorometric assay.

9. Hepatic Na^+/K^+ ATPase activity assay

For the hepatic Na^+/K^+ ATPase activity assay, tissue samples were first homogenized in 10 volumes (1:10, w/v) of a 0.32 mM sucrose solution containing 5 mM HEPES and 1 mM ethylenediaminetetraacetic acid (EDTA) at pH 7.5. The homogenates were then centrifuged, and the supernatants were used for the Na^+/K^+ ATPase activity assay by reacting with 5 mM $MgCl_2$, 80 mM NaCl, 20 mM KCl, and 40 mM Tris-HCl at pH 7.4, in a final volume of 200 μL. After pre-incubation at 37°C, the reaction was initiated by the addition of ATP to a final concentration of 3 mM, and incubated for 20 min. Controls were carried out under the same conditions with the addition of 1 mM of ouabain. The specific activity of Na^+/K^+ ATPase was expressed as nanomoles of inorganic phosphate (Pi) released per minute per milligram of protein.

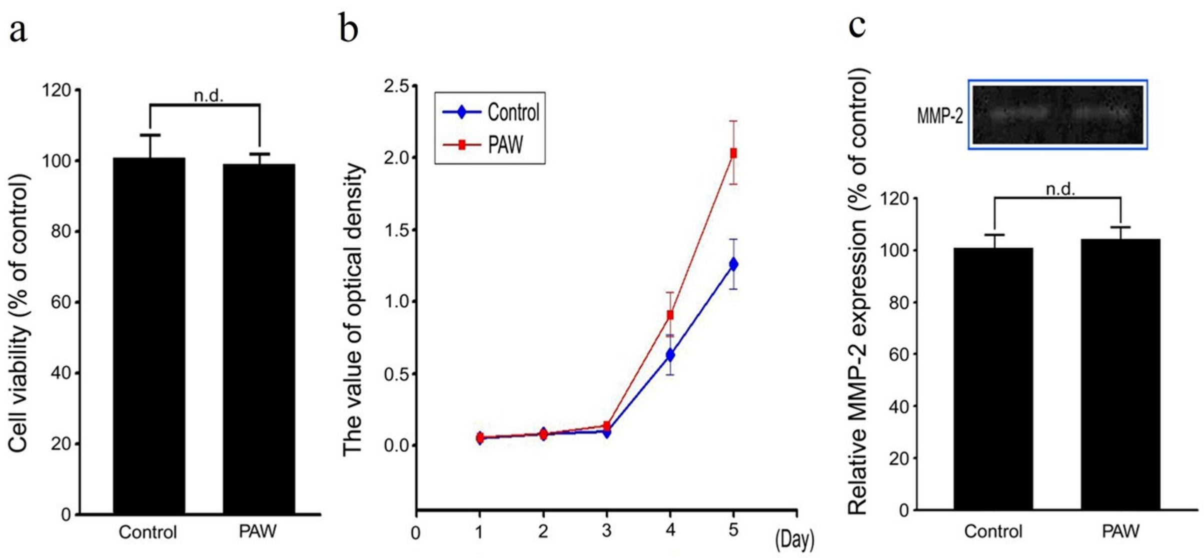

Fig. S1. *In vitro* analysis of the cellular effects of PAW on hepatocytic Clone-9 cells. Note that PAW treatment did not cause cytotoxicity in liver cells as revealed by MTT assay (a). In addition, PAW also effectively enhances cellular proliferation activity (b). Also note that in cells cultured with PAW, no significant difference was detected in MMP-2 (c), suggesting that PAW did not affect cellular migration activity.

References

7 L. Y. Chen, C. Tiong, C. H. Tsai, W. C. Liao, S. F. Yang, S. C. Youn, F. D. Mai and H. M. Chang, *Brain Struc. Funct.*, 2015, **220**, 663-676.

8 A. Sengupta, S. D. Rhoades, E. J. Kim, S. Nayak, G. R. Grant, P. Meerlo and A. M. Weljie, *Int. J. Biochem. Cell Biol.*, 2017, **93**, 129-135.

15 H. C. Chen, B. J. Hwang, F. D. Mai, Y. C. Liu, C. M. Lin, H. S. Kuo, D. S. Chou, M. J. Lee, K. H. Yang, C. C. Yu, J. R. Chen, T. Y. Lo, H. Y. Tsai, C. P. Yang, C. Wang, H. T. Hsieh and J. Rick, *ACS Nano*, 2014, **8**, 2704-2713.

16 H. C. Chen, H. C. Lin, H. H. Chen, F. D. Mai, Y. C. Liu, C. M. Lin, C. C. Chang, H. Y. Tsai and C. P. Yang, *Sci. Rep.*, 2014, **4**, 4425.

17 H. C. Chen, F. D. Mai, K. H. Yang, L. Y. Chen, C. P. Yang and Y. C. Liu, *Anal. Chem.*, 2015, **87**, 808-815.

46 C. C. Yu, Y. C. Liu, K. H. Yang and H. Y. Tsai, *J. Raman Spectrosc.*, 2011, **42**, 621-625.

47 H. M. Chang, C. Y. Tseng, I. H. Wei, J. H. Lue, C. Y. Wen and J. Y. Shieh, *J. Pineal. Res.*, 2005, **39**, 206-214.

48 A. J. Smolen, Image analytic techniques for quantification of immuno-histochemical staining in the nervous system. Conn, P.M., editor, in Quantitative and Qualitative Microscopy, 3, 208-229, Academic Press, San Diego, USA, 1990.
